# The origin of overpotential in lithium-mediated nitrogen reduction†

**DOI:** 10.1039/d2fd00156j

**Published:** 2022-11-29

**Authors:** O. Westhead, R. Tort, M. Spry, J. Rietbrock, R. Jervis, A. Grimaud, A. Bagger, I. E. L. Stephens

**Affiliations:** a Department of Materials, Imperial College London UK i.stephens@imperial.ac.uk; b Department of Chemical Engineering, Imperial College London UK a.bagger@imperial.ac.uk; c Electrochemical Innovation Lab, Department of Chemical Engineering, University College London UK; d Solid-State Chemistry and Energy Laboratory, UMR8260, CNRS, Collège de France France; e Réseau sur le Stockage Electrochimique de l'Energie (RS2E), CNRS FR 3459 80039 Amiens Cedex 1 France; f Department of Chemistry, Merkert Chemistry Center, Boston College Chestnut Hill MA USA

## Abstract

The verification of the lithium-mediated nitrogen reduction system in 2019 has led to an explosion in the literature focussing on improving the metrics of faradaic efficiency, stability, and activity. However, while the literature acknowledges the vast intrinsic overpotential for nitrogen reduction due to the reliance on *in situ* lithium plating, it has thus far been difficult to accurately quantify this overpotential and effectively analyse further voltage losses. In this work, we present a simple method for determining the Reversible Hydrogen Electrode (RHE) potential in the lithium-mediated nitrogen reduction system. This method allows for an investigation of the Nernst equation and reveals sources of potential losses. These are namely the solvation of the lithium ion in the electrolyte and resistive losses due to the formation of the solid electrolyte interphase. The minimum observed overpotential was achieved in a 0.6 M LiClO_4_, 0.5 vol% ethanol in tetrahydrofuran electrolyte. This was −3.59 ± 0.07 V *vs.* RHE, with a measured faradaic efficiency of 6.5 ± 0.2%. Our method allows for easy comparison between the lithium-mediated system and other nitrogen reduction paradigms, including biological and homogeneous mechanisms.

## Abstract 2

We present a simple method for determining the reversible hydrogen electrode potential in a lithium-mediated nitrogen reduction system, and use this to highlight that the greatest source of cathodic potential loss is the requirement for *in situ* lithium plating.

## Graphical abstract

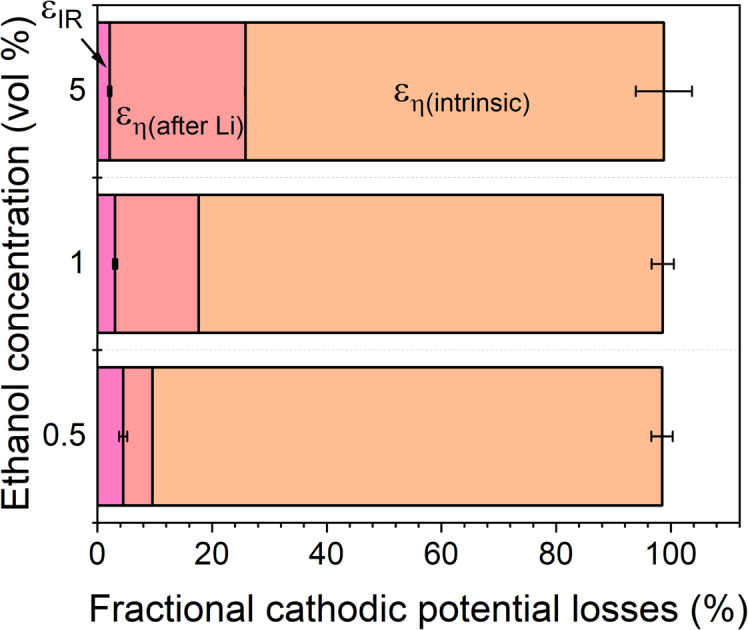

## 1. Introduction

The advent of the Haber–Bosch process in the early 20th century revolutionised the fertiliser industry. Today, approximately 175 million tonnes of ammonia are produced per year with 50% of nitrogen in our bodies originating from Haber–Bosch ammonia.^1,2^ However, the Haber–Bosch process requires high temperatures and pressures (>150 bar, >400 K) to improve kinetics. It also relies on methane-derived hydrogen which, together with the extreme operating conditions, results in an enormous energy consumption and the production of 1% of global CO_2_ emissions.^3^ These operating parameters also necessitate the restriction of ammonia production to large centralised plants to keep capital costs low.^4^ This provides a logistical problem for ammonia delivery to point of use; often farms for use as fertiliser. Indeed, many countries lacking the capital to build a Haber–Bosch ammonia plant and ammonia distribution infrastructure suffer from a lack of ammonia-based fertilisers.^1^ A better solution would be electrochemical ammonia synthesis, which could be carried out at point of use under mild operating conditions using renewable electricity. As yet, the only verified method of electrochemical ammonia synthesis is the non-aqueous lithium-mediated nitrogen reduction system, pioneered by Tsuneto *et al.*^5,6^ and later verified by Andersen *et al.*^7^

The lithium-mediated nitrogen reduction system consists of an organic electrolyte, anhydrous proton donor and lithium salt. Lithium is electrodeposited onto the working electrode *in situ* and creates the active surface for nitrogen reduction. The most common solvent is tetrahydrofuran (THF) and the most common proton donor is ethanol, with different lithium salts yielding varied performance for N_2_ reduction.^8^ LiClO_4_, originally used by Tsuneto *et al.*, is generally outperformed by fluorinated salts such as LiNTf_2_.^9–11^ However, while there have been incredible gains in terms of stability, catalytic activity and selectivity since just 2019,^9,11^ the question of overpotential is rarely addressed. This is in part due to the lack of a suitable non-aqueous reference electrode; most studies use an inert wire pseudo-reference and use the observed lithium plating potential to report their operating potential.

While this potential reporting method is useful for comparing relative electrode potential stability,^8,12,13^ there is no simple way to determine the absolute potential *versus* the Reversible Hydrogen Electrode (RHE) in non-aqueous solvents.^9^ Given that protons are involved in the formation of ammonia from N_2_, the potential for ammonia synthesis is affected by proton activity and has an equilibrium potential measured on the RHE scale. As such, comparing the operating potential in the lithium-mediated system to the thermodynamic equilibrium potential for nitrogen reduction, and therefore to calculate the overpotential, remains a challenge. Given the presence of nitrogen and protons in the electrolyte, it is unlikely that the measured lithium plating potential represents the well defined Li/Li^+^ redox couple. Indeed, the active surface remains uncharacterised, although it is proposed to be some mixed Li*_x_*N*_y_*H_*z*_ complex.^14^ Even the Li/Li^+^ redox couple has been shown to vary (on the order of ∼0.6 V)^15^ in different solvents, and also likely varies in electrolytes containing different salts and proton donors with varying concentrations. These ambiguities complicate the use of the observed lithium plating potential since it is unlikely to represent the Li/Li^+^ redox couple, and even this redox couple is electrolyte dependent.

Variations in electrolyte composition are also likely to change the activity of protons, not only changing the plating potential against the pseudo-reference but also against the RHE potential. This lack of consistency makes it difficult to compare the variations in electrode potential in the different electrolytes used in lithium-mediated nitrogen reduction. In addition, without reporting the lithium-mediated nitrogen reduction operating potential on the RHE scale, it is difficult to accurately compare the system to other nitrogen reduction paradigms such as enzymatic or homogeneous nitrogen fixation.^16^ Although approximations can be made, especially given that the difference in operating potential between lithium-mediated nitrogen reduction and such paradigms is on the order of a few volts,^9^ it would be preferable to avoid such approximations and provide an accurate comparison.

In this work, by examining the behaviour of a hydrogen saturated electrolyte under cyclic voltammetry, we experimentally determine the zero point on a platinum electrode for hydrogen evolution and oxidation before and after ammonia synthesis. This study lays the groundwork for the accurate determination of the overpotential for nitrogen reduction.

## 2. The thermodynamics of lithium-mediated nitrogen reduction

The simplest thermodynamic case would be to consider the formation of gaseous ammonia, especially given that this is ammonia's most stable state,^17^ following the reaction

1 N_2(g)_ + 6H^+^_(solv)_ + 6e^−^ ⇌ 2NH_3(g)_.

However, most reports of electrochemical nitrogen reduction focus on quantifying solvated ammonia, with literature showing that this is where the bulk of the produced ammonia can be found.^7,18^ Generally, the anodic reaction (usually electrolyte oxidation) acidifies the electrolyte, trapping the evolved NH_3_ gas as solvated NH_4_^+^.^19^ It is likely, however, that any contribution from the solvation of NH_3_ in the electrolyte is small, with only a 6 mV anodic shift calculated when considering the reduction potentials for N_2(g)_ to NH_3(g)_*vs.* N_2(g)_ to NH_4_^+^_(solv)_ in acetonitrile.^20^ Therefore, in this study, we consider the reduction of dinitrogen to gaseous ammonia to determine the thermodynamic equilibrium potential for nitrogen reduction.

The Nernst equation states that the standard Gibbs free energy of formation of a substance in an electrochemical reaction, Δ*G*^0^, is related to the equilibrium potential for the reaction under standard conditions, *U*^0^, by

2 Δ*G*^0^ = −*nFU*^0^,

where *n* is the number of electrons involved in the reaction and *F* is the Faraday constant. For eqn (1), given that the standard Gibbs free energy of formation of protons and N_2_ is zero, we can write3
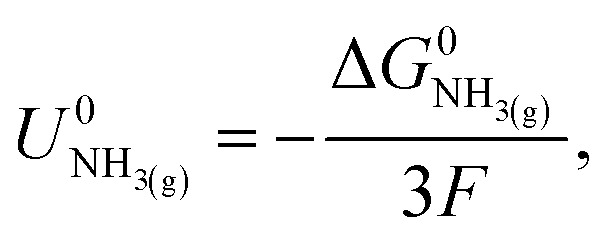
where Δ*G*_NH_3(g)__ is the standard molar Gibbs energy of formation of ammonia and *F* is the Faraday constant. Since 
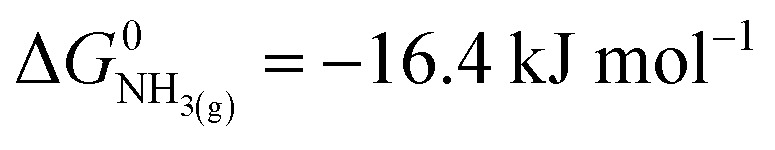
,^21^ we find that 
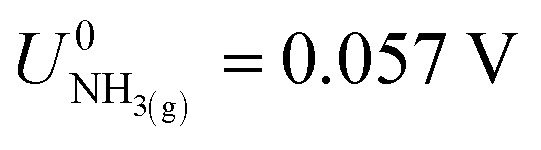
*vs.* RHE.

However, the lithium-mediated nitrogen reduction system does not directly produce ammonia over a pre-made metal catalyst. Rather, the electrodeposition of lithium *in situ* catalyses the reaction. In addition, the exact mechanism for lithium-mediated nitrogen reduction is as yet unknown. There are various proposed mechanisms,^3,13,14^ some considering lithium as a pure metallic surface^13^ and others considering the formation of bulk phases such as lithium hydride or lithium nitride.^14^ However, in all cases, lithium deposition potentials are required to provide an active catalyst.

## 3. Considering the active electrode

Given the presence of protons and nitrogen in the electrolyte for lithium-mediated nitrogen reduction, the active electrode is likely to consist of lithium, lithium nitride, lithium hydride or some mixed combination of lithium, nitrogen, and hydrogen (Li_*x*_N_*y*_H_*z*_).^14^ Post-mortem characterisation of the working electrode is yet to unequivocally characterise nitrogen containing active catalyst species, which is likely due to the strong thermodynamic driving force for such a species to be protonated to ammonia.^8,14^ However, the nature of the active catalyst affects the reduction mechanism, and thus the thermodynamics, for ammonia production.

All reduction schemes considered in the literature^3,13,14^ rely on lithium metal to form the active surface, and so it is important to consider the potential required for lithium plating. From the Nernst equation, the potential for lithium plating depends on the activity of lithium ions in solution and is written as4
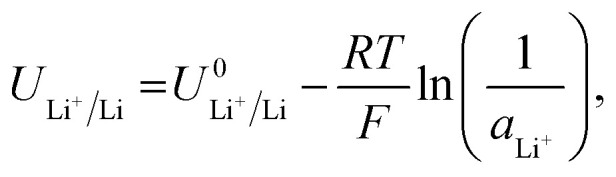
where 
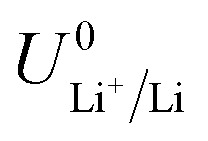
 is the equilibrium potential for lithium plating, *R* is the ideal gas constant, *T* is temperature and *a*_Li^+^_ is the chemical activity of lithium-ions. Clearly, therefore, the use of different salts, salt concentrations, and solvents, which affect lithium-ion activity, will affect the lithium plating potential.

The standard potential for lithium plating, 
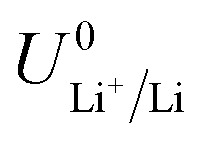
, also varies in different solvents.^22^ In water, the standard potential is −3.04 V *vs.* the Standard Hydrogen Electrode (SHE), which translates to −2.63 V *vs.* RHE assuming a pH of 7.^23^ In pure ethanol and tetrahydrofuran, the standard reduction potentials are −3.02 V and −2.98 V *vs.* SHE respectively.^23^ It has been shown that the absolute potential for lithium plating is related to the solvation of the lithium ion in the electrolyte *via* the relation5
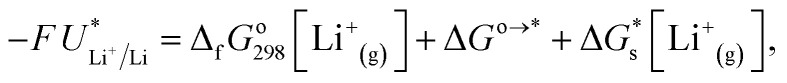
where Δ_f_*G*^o^_298_[Li^+^_(g)_] and Δ*G*^o→^* are solvent-independent variables relating to gas phase ionization and standard state correction respectively, and 
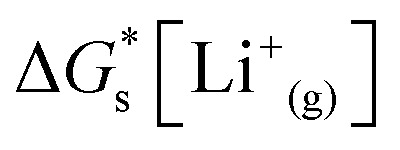
 is the solvation free energy of the lithium-ion.^24^ The solvation free energy of lithium ions is dependent on the salt anion and solvent donor number, with higher donor number anions resulting in more Li^+^ ion–anion interactions and higher donor number solvents resulting in fewer Li^+^ ion–anion interactions.^25^

While in batteries this electrolyte dependent shift in 
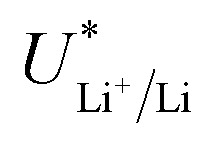
 is counterbalanced by the equal shift in potential on the positive electrode and so does not affect the energy efficiency of the full cell, the anodic reaction potential in the lithium mediated system is unlikely to vary in the same way as the cathodic reaction. Most reported systems do not control the anodic reaction, which results in solvent oxidation.^19,26^ The bulk of the electrolyte considered in this work is THF, and so for simplicity we will consider just the oxidation potential of THF. The one electron oxidation of THF is written

6 THF˙^+^_(solv)_ + M(e^−^) ⇋ THF_(l)_,

where M(e^−^) is a metallic electron.^27^ The free energy for reaction (6) can be written

7 Δ*G*_THF/THF˙^+^_ = −Δ*G*_vap_ + *Φ*_M_ − Δ*G*_e_(THF) + Δ*G*_solv_(THF˙^+^),

where Δ*G*_vap_ is the free energy of solvent vaporisation, Δ*G*_e_(THF) is the free energy of ionisation of the THF molecule, Δ*G*_solv_(THF˙^+^) is the solvation energy of the radical cation THF˙^+^ and *Φ*_M_ is the work function of the metal electrode, where the sign convention is that all thermodynamic quantities are absolute values.^27^ The standard reduction potential *vs.* a given reference system with free energy change Δ*G*_ref_ is then written, from eqn (2),8
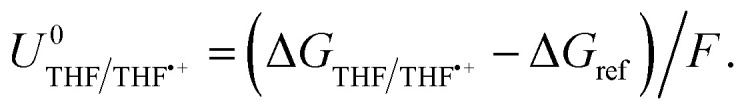


The oxidation potential of THF is also affected by the presence of lithium salt but is not dependent on the activity of the lithium-ion. Instead, oxidation of the salt anion may occur which, in THF in particular, can result in polymerization of the THF which may shift the potential.^28^ Thus, the oxidation potential of THF does not depend on the same parameters as the lithium plating potential, and so any shift in lithium plating will not be counterbalanced by a similar shift in oxidation potential. If the Hydrogen Oxidation Reaction (HOR) were used as the anodic reaction, the anode potential would depend solely on proton activity in the electrolyte, whereas the Li/Li^+^ potential would also be affected by the aforementioned parameters in eqn (4) and (5). However, no matter the shift in the lithium redox potential, the need to reduce Li^+^ ions will always result in a large overpotential for nitrogen reduction when compared to the equilibrium potential given by eqn (3).

The potential for nitrogen reduction over the lithium surface is given by9
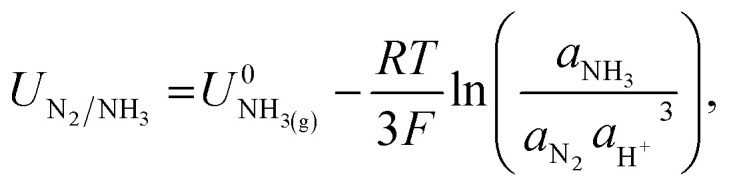
where *a*_x_ is the activity of substance x. For operation under ambient conditions, as in this work, we assume the partial pressure of N_2_ is 1 bar, which means *a*_N_2__ is 1. Therefore, the overpotential for nitrogen reduction over a lithium surface depends only on *a*_NH_3__ and *a*_H^+^_; the activity of ammonia and protons respectively. However, given the extreme lithium plating potentials already applied, any correction to the nitrogen reduction potential due to proton or ammonia activity is negligible.

It is also important to consider the formation energies required for other electrodes, such as lithium hydride. The formation of lithium hydride from metallic lithium and protons can be written

10 Li_(s)_ + H^+^_(solv)_ + e^−^ ⇌ LiH_(s)_.

Similarly to eqn (3), the standard Gibbs free energy of protons and pure lithium in the condensed phase are zero. The standard Gibbs free energy of formation of lithium hydride in the condensed phase is Δ*G*_LiH_(s)__ = −68.3 kJ mol^−1^,^21^ and so the standard potential for lithium hydride formation on a lithium surface can be written11
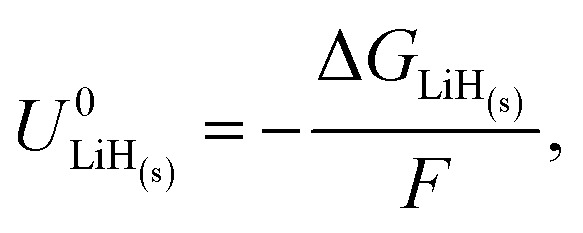
which results in a potential of *U*^0^_LiH(s)_ = −0.708 V *vs.* RHE. The potential for lithium hydride production will depend on the activity of protons by the Nernst equation12
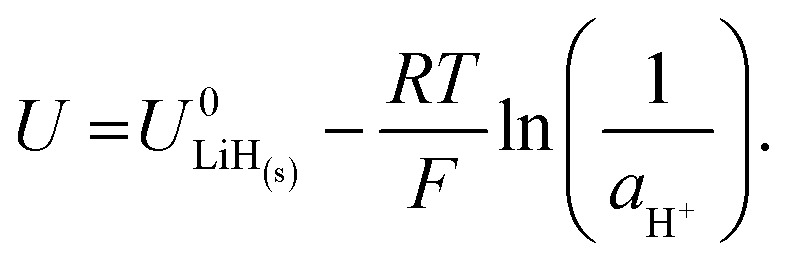
Thus, in the presence of protons, there is a strong driving force to form lithium hydride at lithium plating potentials, assuming that not all protons are consumed by hydrogen evolution.

Lithium nitride has a Gibbs energy of formation of Δ*G*_Li_3_N_(s)__ = −154.8 kJ mol^−1^ (−1.6 eV) at room temperature^29^ and is formed chemically. Therefore, as Schwalbe *et al.* note, there is a strong driving force to form both LiH and Li_3_N at lithium plating potentials. The same authors calculate *via* Density Functional Theory (DFT) that all proton coupled electron transfers towards ammonia formation will be exergonic at potentials negative of −0.76 V *vs.* RHE on Li, −1.33 V *vs.* RHE on Li_3_N and −1.53 V *vs.* RHE on LiH surfaces.^14^ Thus, a lithium surface is predicted to be the most active for nitrogen reduction. DFT calculations also suggest that only metallic lithium is able to perform adsorption and dissociation of N_2_.^30^ Increasing the activity of protons in the electrolyte is likely to result in a greater quantity of lithium hydride, which may unfavourably shift the thermodynamics as well as reducing faradaic efficiency to nitrogen reduction and promoting hydrogen evolution.^9,31^ However, at the extremely cathodic potentials required to reduce Li^+^, we consider all charge transfer steps to be extremely exergonic, except for Li plating itself.

## 4. Measuring the RHE potential

The RHE potential is based upon the reversible oxidation and reduction of protons and molecular hydrogen, written as

13 2H^+^ + 2e^−^ ⇌ H_2(g)_.

By convention, the standard potential for hydrogen oxidation and reduction is 0 V *vs.* RHE, regardless of the activity of protons. Hence, from the Nernst equation the RHE potential (*U*_RHE_) is given by the standard potential and the activity of protons as14
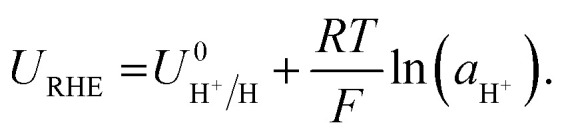


The ideal experiment to measure the RHE potential for nitrogen reduction would require a well-defined reference electrode which is inert under hydrogen and stable in the organic electrolyte under the extreme potentials required for lithium plating. Such a reference electrode is difficult to find for non-aqueous electrochemistry. Since acceptance of this manuscript, we have developed a stable reference electrode based on a half-lithiated battery cathode material, Lithium Iron Phosphate (LFP).^32–34^ This has been shown to have a stable open circuit even under oxygen and hydrogen atmosphere.^32–34^ Previous work evaluating hydrogen redox has used an Ag wire pseudo-reference calibrated against the ferrocene redox couple.^19^ However, our initial experiments show that an Ag wire pseudo-reference was not stable in a 0.2 M LiBF_4_ 1% vol ethanol in THF electrolyte under nitrogen reduction conditions (see ESI Fig. S1†). Such measurements are necessary to determine the overpotential for nitrogen reduction. Therefore, the tests in this work were carried out using a Pt wire pseudo-reference which has been shown to be reliably stable against dissolution.^8,35^

**Fig. 1:**
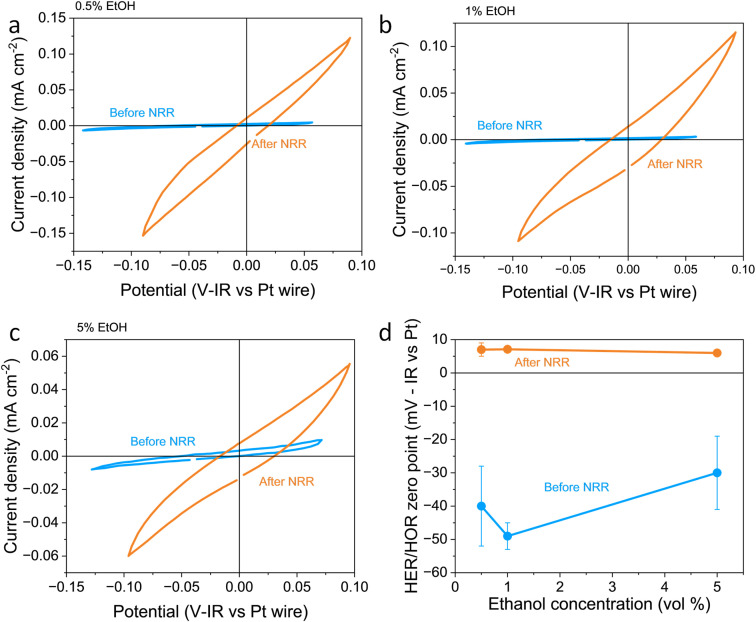
Plots to show the variation in reversible hydrogen electrode potential and hydrogen evolution and oxidation activity with ethanol content before and after a nitrogen reduction experiment. The electrolyte was 0.6 M LiClO_4_ in THF with varying ethanol content. The working electrode was a Pt foil, the counter electrode was a Pt mesh of higher surface area than the working electrode, and the reference was a Pt wire pseudo-reference. (a–c) show a representative fifth cyclic voltammogram from a single experiment taken at 20 mV s^−1^ under 1 bar hydrogen partial pressure before nitrogen reduction (in blue) and after nitrogen reduction (in orange) for 0.5, 1 and 5 vol% ethanol respectively. (d) shows the variation in the hydrogen evolution reaction (HER)/hydrogen oxidation reaction (HOR) zero-point potential before and after nitrogen reduction averaged for *n* = 3 separate experiments. Error bars smaller than points for 1 and 5 vol% ethanol after NRR. See ESI† for full experimental details.

In this work, the RHE potential was measured *vs.* the Pt pseudo-reference before and after a nitrogen reduction experiment in which −5 C were passed at a constant applied current density of −2 mA cm^−2^. Fig. 1a–c show how the activity and measured potential vary before and after the nitrogen reduction experiment with varying ethanol content in a 0.6 M LiClO_4_ in THF electrolyte across a platinum foil working electrode. In all experiments, regardless of ethanol content, the measured Hydrogen Evolution Reaction (HER) and HOR activity was much higher after a nitrogen reduction experiment than before. Some change in activity around 0 V *vs.* Pt wire can even be seen in the absence of H_2_ (see Fig. S2a†). However, there was activity which we assign to HER/HOR at around 0 V *vs.* Pt wire which was not seen in Ar both before and after nitrogen reduction (see Fig. S2b and c†). This is in line with previous studies in the literature which reported electrolyte acidification across the course of an experiment;^19^ after nitrogen reduction, there is a greater proton activity due to solvent oxidation occurring at the counter electrode. Fig. S2d† shows that there is no significant relationship between hydrogen evolution activity and ethanol concentration, but all experiments show an increase in activity after nitrogen reduction. This significant change in proton activity which seems unaffected by ethanol concentration brings the role of ethanol, ostensibly the proton donor, into dispute. Indeed, Du *et al.*^11^ note greater levels of ammonia production than the concentration of ethanol in their electrolyte should allow. This observation, along with this work, suggests that proton activity is not driven solely by ethanol concentration.

Fig. 1d also shows the measured HER/HOR zero point before and after a nitrogen reduction experiment for the three ethanol concentrations considered. Before nitrogen reduction, there may be an increase in the zero point with ethanol content, but the errors on the data points are too large to be certain. After nitrogen reduction, there is no real change in zero point with ethanol concentration. The higher level of uncertainty in the data points before nitrogen reduction is likely due to the very low HER/HOR activity. Platinum has been shown to suffer from poisoning by organic solvents such as acetonitrile, which can cause voltage losses in a fuel cell, with poisoning effects being more extreme at lower current densities.^36^ This poisoning can occur due to organic oxidation products being present on the platinum electrode. In addition, trace water in organic electrolytes has been shown to affect the reversibility of the HER/HOR redox couple since protons generated by hydrogen oxidation are strongly solvated by trace water and carried away from the electrode surface.^37^ It makes sense that this effect would be worsened at lower HER/HOR activities, since the ratio of trace water to evolved protons would be higher. As such, the uncertainty in the measurements before nitrogen reduction at lower HER/HOR activities is higher.

A shift in the measured RHE potential with respect to the Pt wire before and after nitrogen reduction can also be observed. This may be due to an increased proton activity, following the Nernst equation (eqn (14)), but given that the reference electrode used in this case was also platinum and thus the open circuit potential between working and reference electrodes should theoretically be zero, it is likely that the effect of impurities and poisoning played a greater role in the less active condition. Therefore, the value obtained after the nitrogen reduction experiment was used for further calculations.

The change in proton activity before and after a nitrogen reduction experiment, as previously noted by Krempl *et al.*^19^ and observed in this work, presents a difficulty for determining the RHE potential across a whole experiment. A similar problem is faced in the field of aqueous CO_2_ reduction, where the pH at the working electrode interface has been shown to vary over the course of a reaction.^38^ Use of the bulk pH to convert to the RHE scale may cause underestimation of the true overpotential for some reactions.^38^ In the case of non-aqueous nitrogen reduction, where pH is not well defined, we instead consider the change in proton activity in the electrolyte. Given that in this work the electrolyte was stirred, proton activity changes will not be completely restricted to the electrode surface. However, a change in bulk proton activity over the course of an experiment will alter the zero point for the RHE potential as measured by hydrogen oxidation and evolution. One way of avoiding this issue could be to calibrate a stable reference electrode against RHE in a solution of known proton activity, and then use the calibrated reference electrode to obtain the nitrogen reduction operating potential.

## 5. Measuring nitrogen reduction overpotential

**Fig. 2:**
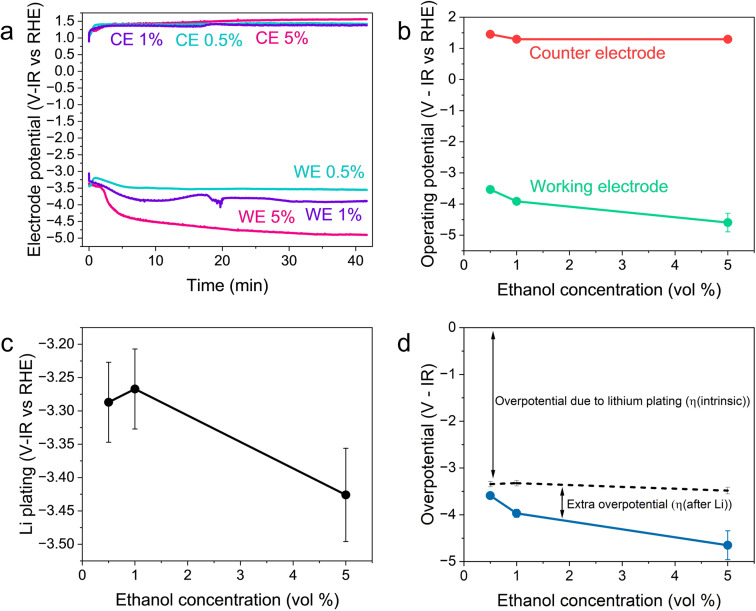
Plots to show the variation in operating potential and lithium plating potential during nitrogen reduction. After a 1 hour purge with N_2_ gas at 10 ml min^−1^ to remove any dissolved H_2_ gas, a linear sweep voltammogram was taken at 20 mV s^−1^ to determine the lithium plating potential and then a current density of −2 mA cm^−2^ was applied until −5 C of charge had passed. A molybdenum foil working electrode, Pt wire pseudo-reference and Pt mesh counter electrode were used. The electrolyte was 0.6 M LiClO_4_ in THF with varying concentrations of ethanol added. The thermodynamic equilibrium potential for nitrogen reduction is 0.057 V *vs* RHE. All potentials plotted are IR corrected. (a) Three representative chronopotentiometry experiments taken at −2 mA cm^−2^. (b) The average operating potentials across a chronopotentiometry experiment under an applied current density of −2 mA cm^−2^ for three different ethanol concentrations (*n* = 3). (c) The variation in lithium plating potential observed *via* linear sweep voltammetry (*n* = 3). For (a–c), the voltages are corrected for ohmic drop and converted to the measured RHE potential using the average values obtained from three separate experiments after a nitrogen reduction experiment, since these exhibited less variation (see Fig. 1d). (d) The calculated overpotential for nitrogen reduction, not including ohmic drop. The blue line represents the total overpotential. The dashed line represents the overpotential due to lithium plating (η(intrinsic)). The difference between the blue line and dashed line represents any extra overpotential after lithium plating (η(after Li)).

Fig. 2 shows the variation in the operating potentials during nitrogen reduction and lithium plating potentials with ethanol content against the measured RHE potential after nitrogen reduction from Fig. 1d. The hydrogen measurements before and after nitrogen reduction did not appear to adversely affect nitrogen reduction, since the obtained faradaic efficiencies and yield rates are comparable to those obtained without hydrogen measurements (see Fig. S3†). Fig. 2a and b show a clear dependence of nitrogen reduction operating potential on ethanol concentration, with the operating potential becoming more negative with increasing ethanol content by approximately 1 V. Fig. 2c shows that the lithium plating potential also appears to depend on ethanol concentration, although uncertainty is higher in these measurements. The effect is also less strong, with a change in only approximately 0.2 V observed. The operating potential also varies against the observed lithium plating potential, as shown in Fig. 2d. It is clear from these figures that the overpotential required for lithium-mediated nitrogen reduction is enormous.

Fig. 2a and b show less of an impact of ethanol concentration on the counter electrode potential. The counter reaction is likely to be solvent oxidation. The oxidation potential of THF is reported as around 4.2 V *vs.* Li^+^/Li.^27,39^ From the measurements of the lithium plating potential *vs.* RHE herein, this would put the THF oxidation potential at around 0.85 V *vs.* RHE in this electrolyte, depending on the ethanol concentration. Ethanol begins to oxidise at around 0.4 V *vs.* RHE on platinum.^19,40^ Our experiments show solvent oxidation beginning at around 0.6 V *vs.* the Pt pseudo-reference in argon for the 1 vol% ethanol condition (Fig. S4†), which would correspond to around 0.53 V *vs.* RHE. There are various oxidation products that can be formed such as acetaldehyde, which can be further oxidised to acetic acid, which has been detected in literature,^26^ or CO gas and other fragments.^40^ The counter electrode potential in Fig. 2a is relatively stable between 1.3 and 1.5 V *vs.* RHE, and does not vary significantly with ethanol concentration (Fig. 2b). This value is well above the approximate oxidation potentials for both THF and ethanol. Thus, both THF and ethanol are oxidised during this process^26^ but, given that the bulk of the solvent is THF, the increase in ethanol content is not sufficient to produce a significant change in counter electrode potential.

As discussed earlier, variation in the solvent will result in a change in the plating potential (eqn (4) and (5)). Fig. 2c shows a decrease in the observed lithium plating potential with increasing ethanol content. This may result from a change in the solvation of the lithium ions in the mixed electrolyte, with increased ethanol content changing the solvation free energy of the lithium ions in solution. Thus, it is likely that increasing the concentration of ethanol in the electrolyte displaces the equilibrium due to a stronger solvation of lithium-ions, pushing the lithium plating potential to more negative values.^15^ A similar effect can be seen with variation in LiClO_4_ concentration at a constant ethanol concentration. Fig. S5† shows the variation in lithium plating potential *vs.* the Pt wire pseudo-reference with LiClO_4_ concentration in a 1 vol% ethanol in THF electrolyte. Here, the measured potential for lithium plating becomes more positive with increasing salt concentration. This phenomenon has also been linked to lithium-ion solvation in an electrolyte, with the formation of different solvation structures in the electrolyte resulting in plating potential shifts.^15^ However, it seems counter-intuitive that, given that the bulk of the electrolyte is still THF, the change in ethanol concentration alters the solubility of lithium-ions so much to account for the entirety of this shift. Indeed, the shift from a pure THF to a pure ethanol electrolyte would only result in a shift of 0.04 V *vs.* SHE.^23^

It may also be that changing the concentration of ethanol in the electrolyte alters the activity of protons in the electrolyte, changing the RHE potential according to eqn (14). This alters the reference potential, thus changing the measured lithium plating potential *vs.* RHE. Given that the ethanol concentration, ostensibly the source of protons, was varied across an order of magnitude, it would be reasonable to assume that some effect would be observed in the RHE potential. However, given the similar activities observed during HER/HOR redox (Fig. 1 and S2d†) for all ethanol concentrations considered, it would appear that ethanol concentration is not the main driver of proton activity. Instead, solvent oxidation acidifies the electrolyte and likely provides the bulk of the protons^19^ (see Fig. S2†). As previously discussed, both ethanol and THF are likely oxidised at the anode. Gas Chromatography Mass Spectrometry (GCMS) analysis reveals that the bulk of the oxidation products at the anode in a LiClO_4_ based electrolyte with 1% v/v ethanol in THF are from THF oxidation. The main products observed were 4-hydroxybutanal or tetrahydrofuran-2-ol, which are difficult to distinguish between.^26^ Both products result from THF oxidation. Acetic acid is observed from ethanol oxidation, but in much smaller quantities (approximately an order of magnitude).^26^ It is therefore unlikely that the increase in ethanol content would alter the proton activity so much to account for the full observed ∼−0.2 V shift in measured lithium plating potential *vs.* RHE. From eqn (14), if this negative shift were just due to a change in proton activity affecting the RHE potential, the proton activity from 0.5 to 5 vol% ethanol would need to decrease approximately 2000-fold.

Thus, the role of ethanol is complex in lithium-mediated nitrogen reduction. The greatest effect of ethanol concentration on the system may lie in the formation of the Solid Electrolyte Interphase (SEI), rather than in the ostensible role as a sacrificial proton donor.^31,42^ Indeed, Lazouski *et al.* note that rather than a volcano like relationship between proton donor p*K*_a_ and nitrogen reduction faradaic efficiency, which may be intuitively expected, there is instead only a sharp peak in efficiency in a narrow region between 15 and 17.5, which are all simple alcohols.^31^ In addition, Fu and Pedersen *et al.* recently showed that ethanol plays a role as a proton shuttle. By using the HOR as a counter reaction and employing deuterated hydrogen as an anodic feed gas, the authors show a shift to the production of deuterated ammonia over the course of an experiment.^41^ The SEI, which provides kinetic stability from continued electrolyte decomposition to the system under the highly negative operating potentials,^43^ is formed from the initial decomposition products of the electrolyte and salt. Recent literature has shown that the content of the SEI has a significant impact on the stability as well as the faradaic and energy efficiency of the system.^8,10,11,35^ It may be that the change in ethanol content impacted the transport of lithium through the SEI, which may have affected the lithium plating potential and operating potential. Indeed, cryogenic Transmission Electron Microscopy (cryo-TEM) and Scanning Electron Microscopy (SEM) investigations may reveal very different SEI morphologies in the presence of ethanol and without.^42^ The SEI in an ethanol containing electrolyte is generally more porous and less passivating than the same electrolyte without ethanol.^44^ Thus, it may be that the different SEI properties gained *via* the presence of ethanol activate the surface for nitrogen reduction, rather than ethanol simply acting as a relatively inert proton donor.^42^

To consider the effect of lithium-ion transport through the SEI, we can use the Doyle–Fuller–Newman (DFN) model of lithium-ion batteries.^44^ Here, the overpotential for lithium plating, *η*_Li_, is written

**Fig. 3:**
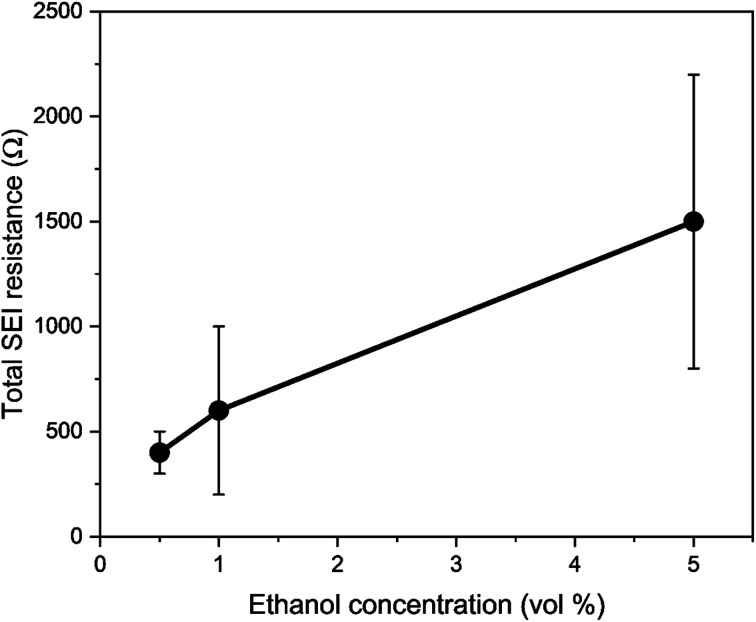
A plot to show the variation in total solid electrolyte interphase (SEI) resistance (charge transfer resistance (*R*_CT_) + SEI resistance (*R*_SEI_)) with ethanol concentration. Resistance values were obtained using potentiostatic electrochemical impedance spectroscopy (PEIS). Spectra were taken at open circuit potential after a nitrogen reduction experiment, which is at lithium plating potentials, at an amplitude of 10 mV between 200 kHz and 200 mHz. The total resistance is the sum of the resistance of the two observed semi-circles representing charge transfer resistance and SEI resistance. In general, increasing ethanol content leads to a higher total resistance (*n* = 3).

15 *η*_Li_ = *φ*_s_ − *φ*_e_ − *η*_SEI_,

where *η*_SEI_ is the overpotential related to the SEI and *φ*_s_ and *φ*_e_ are the solid and liquid phase potentials respectively. *η*_SEI_ can be related to SEI properties by16
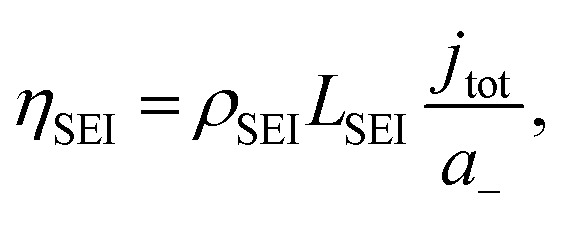
where *ρ*_SEI_ and *L*_SEI_ are the resistivity and thickness of the SEI respectively, *j*_tot_ is the interfacial current density and *a*_−_ is the surface area to volume ratio of the negative electrode.^44^Fig. 3 shows that the total interfacial resistance as measured by Potentiostatic Electrochemical Impedance Spectroscopy (PEIS) generally increases with ethanol content, although there was significant variation in the resistances obtained. The thickness of the obtained SEI was not measured, but recent work suggests that this also varies with ethanol content since initial cryo-TEM analysis suggests that the SEI is thicker in the presence of ethanol than without.^42^ Therefore, considering Fig. 1 and recent microscopy studies,^42^ it appears that the ethanol content has a greater effect on SEI composition, morphology, and resistance than on proton activity in the electrolyte. Many other studies^8,10,35^ note the impact of SEI composition and resistance on faradaic efficiency, which also varies with ethanol content (Fig. S3†).^3,5^ It appears, therefore, that the true role of ethanol requires greater interrogation.

Therefore, a combination of factors relating to SEI resistance and thickness and the solvation of the lithium ion, as well as perhaps a small change in the proton activity changing the RHE potential, resulted in the overall decrease in lithium-plating potential *vs.* RHE with an increase in ethanol content. Indeed, Fig. 2a shows that although the working electrode potentials begin the chronopotentiometry measurement at or close to the measured lithium plating potential, the operating potential at −2 mA cm^−2^ settles at a more negative value, despite the near negatively infinite gradient for current density with potential after lithium plating (see Fig. S5†). It is likely that the SEI is not fully formed at the beginning of the chronopotentiometry measurement, and so it may be that increasing SEI resistance causes the more negative operating potentials in analogy to previous studies.^8^

Fig. 2d shows the variation in measured overpotential for each of the paradigms investigated. The 0.5 vol% ethanol experiments resulted in the highest faradaic efficiency (Fig. S3†) and the lowest overpotential of −0.19 ± 0.07 V *vs.* RHE more negative than the intrinsic overpotential resulting from the requirement for lithium plating. From these values, we can estimate the voltage efficiency (*η*_*U*_) of the cathodic reaction, given by17
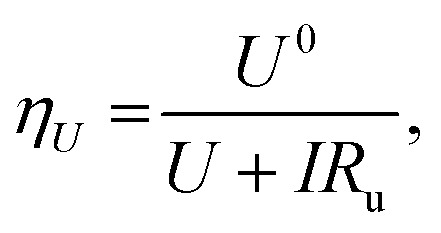
where *IR*_u_ represents the ohmic losses due to the uncompensated electrolyte resistance, *R*_u_. The voltage efficiencies are 1.54 ± 0.02%, 1.41 ± 0.03% and 1.21 ± 0.08% for the 0.5, 1 and 5 vol% ethanol conditions respectively. It is clear that the requirement for lithium plating pushes the total voltage efficiency to untenable values. However, aside from the lithium plating the other sources of overpotential are small in comparison.

**Fig. 4:**
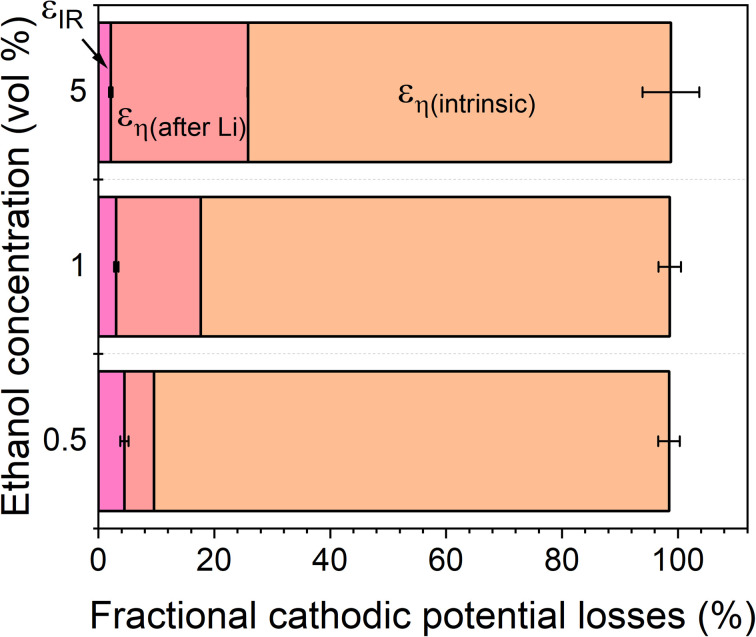
A plot to show the fractional potential losses in the cathodic reaction for the lithium-mediated nitrogen reduction system due to overpotential and ohmic losses for a 0.6 M LiClO_4_ in THF electrolyte with varying ethanol content. Fractional energy losses obtained using eqn (18) and (19).

We can further highlight this by deconvoluting the fractions of energy lost to overpotentials (*ε*_*η*_) and uncompensated resistance (*ε*_*IR*_u__). These are written18
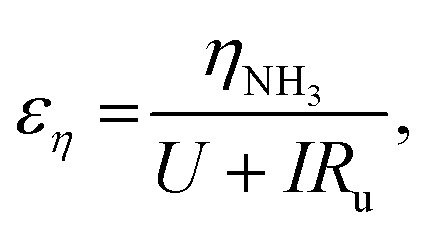
and19
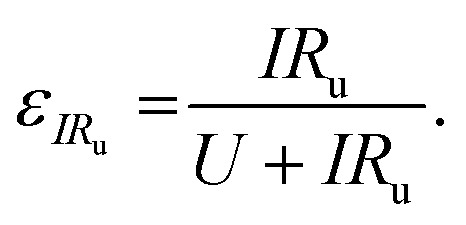
*η*_NH_3__ can be further deconvoluted into the intrinsic overpotential due to the requirement for lithium plating (*η*_intrinsic_), which is given by the observed lithium plating potential *vs.* RHE, and the extra overpotential after lithium plating (*η*_after Li_). Fig. 4 shows how variation in ohmic losses and overpotential affect the distribution of energy losses in the lithium-mediated nitrogen system. In all cases, the potential losses due to overpotential dominate. The intrinsic overpotential plays the greatest role in the 0.5 vol% ethanol condition due to the much lower overpotential after lithium plating. However, it is still the dominating source of cathodic potential loss in the system for all conditions.

The total energy efficiencies for this work are 1.5 ± 0.2%, 1.4 ± 0.1% and 0.55 ± 0.05% for the 0.5, 1 and 5 vol% ethanol cases respectively (see ESI† for energy efficiency calculations). Although, as shown in Fig. 4, the bulk of the cathodic potential losses originate from the requirement for *in situ* plated lithium, the use of an uncontrolled counter reaction (solvent oxidation) also results in potential losses. The anodic potential in this work sits at around 1.5 V *vs* RHE (Fig. 2a). If the HOR were to be used instead, the anodic potential would be much closer to 0 V *vs* RHE, which would reduce the total cell potential. Assuming an anodic potential of 0.4 V *vs* RHE instead, running an HOR reaction, this would reduce the cell potential by approximately 1.1 V. However, Fig. 2(c and d) show us that the intrinsic overpotential due to the requirement of lithium plating is over 3 V. Thus, the greatest source of overpotential in the lithium mediated nitrogen reduction paradigm is lithium plating.

## 6. Conclusion

In this work we propose a simple method for estimating the RHE potential in the lithium-mediated nitrogen reduction system. It would be trivial to apply this method using a more stable and well defined non-aqueous reference electrode.

This work also highlights the issue of changing proton activity throughout a lithium-mediated nitrogen reduction experiment in a single compartment set-up, as originally noted by Krempl *et al.*^19^ We show that the lack of proton activity in the fresh electrolyte leads to significant error in the measurement of the zero point for HER/HOR redox, likely due to a greater impact of platinum electrode poisoning by the organic electrolyte^36^ and trace water contamination altering HER/HOR reversibility.^37^ After a nitrogen reduction experiment, the oxidation of the electrolyte at the anode leads to a much greater proton activity in the electrolyte which reduces error in the determination of the RHE potential. It also appears that, similar to what was observed in the literature,^19^ rather than proton activity being driven by ethanol concentration, solvent oxidation plays a greater role. This brings the true role of ethanol into dispute. While the presence of a proton donor has been repeatedly shown to be necessary,^31^ perhaps it plays a larger role in SEI formation, as shown in this work.

Through the determination of the RHE potential, it is also possible to determine the voltage efficiency of the system. Although there have been attempts to quantify the energy efficiency of lithium-mediated nitrogen reduction,^3,13,18^ such values are difficult to determine without determination of the overpotential on the RHE scale. In this work, we calculate the voltage efficiency and show, unsurprisingly, that the dominating source of energy loss is the vast intrinsic overpotential due to the requirement of lithium plating. Clearly, a system relying on lithium plating is challenged economically, especially for the use of ammonia as a carbon-free fuel where potential losses play a greater role.^45^ We hope that this work will motivate further discussions on overpotential reduction and the possibility of finding an active nitrogen reduction surface beyond lithium.

## Conflicts of interest

There are no conflicts to declare.

## Supplementary Material

FD-243-D2FD00156J-s001
