# Supplementary material for: The origin of overpotential in lithium-mediated nitrogen reduction
Source: Faraday Discuss. 2022 Nov 29;243:321–38. doi: 10.1039/d2fd00156j (PMC10354832; doi:10.1039/d2fd00156j)
Supplement: FD-243-D2FD00156J-s001 [file FD-243-D2FD00156J-s001.pdf]

Electronic Supplementary Material (ESI) for Faraday Discussions.
This journal is © The Royal Society of Chemistry 2023

Supplementary Information for: The Origin of Overpotential in Lithium-Mediated Nitrogen Reduction

O. Westhead[1], R. Tort[2], M. Spry[1], J. Rietbrock[1], R. Jervis[3], A. Grimaud[4,5,6] , A. Bagger[2]*, I.E.L. Stephens[1]*

[1]Department of Materials, Imperial College London

[2]Department of Chemical Engineering, Imperial College London

[3] Electrochemical Innovation Lab, Department of Chemical Engineering, University College London

[4]Solid-State Chemistry and Energy Laboratory, UMR8260, CNRS, Collège de France

[5]Réseau sur le Stockage Electrochimique de l'Energie (RS2E), CNRS FR 3459, 80039 Amiens Cedex 1, France

[6]Department of Chemistry, Merkert Chemistry Center, Boston College, Chestnut Hill, MA

*email: a.bagger@imperial.ac.uk, i.stephens@imperial.ac.uk

1 – Materials

2 – Electrochemical cell preparation

3 – Electrochemical testing

4 – HER/HOR determination

5 – Ammonia quantification

6 – Energy efficiency calculations

1 – Materials

Tetrahydrofuran (anhydrous, ≥99.9%, inhibitor-free), hydrochloric acid (HCl) (30%, Suprapur) and phenol nitroprusside solution were purchased from Sigma-Aldrich. Anhydrous lithium perchlorate (99%, anhydrous) was purchased from Alfa Aesar. Sodium hypochlorite (14% Cl_2 in aqueous solution, GPR RECTAPUR) and sodium hydroxide (pellets AnalaR NORMAPUR) were purchased from VWR. Ethanol (99.9%, Extra Dry, AcroSeal) and nitric acid (HNO_3) (70%, analytical reagent grade) were purchased from Fisher Scientific. Platinum mesh (wire diameter 0.1mm, nominal aperture 0.4 mm, purity 99.9%), Platinum wire (diameter 0.5 mm, 99.99%, as drawn), Platinum foil (0.125 mm thick, as rolled, 99.99%), molybdenum foil (0.125 mm thick, annealed, 99.9%) and copper wire (0.5 mm, 99.99%, as drawn) were purchased from Goodfellow Cambridge. Single compartment glass cell was custom made by Artistic and Scientific Glassware, Oxford. Purifiers for the Ar and N_2 gas lines providing purity levels of H_2O, H_2, CO_2, O_2, CO, nonmethane hydrocarbon (NMHC), CH_4, NH_3, NO_x to < 0.5 ppb were purchased from NuPure. N6 Ar, N6 N2 and N5.5 H2 gas were purchased from BOC. Electrochemistry and electrolyte preparation was carried out in an Ar atmosphere glovebox (MBraun, H_2O <0.1 ppm, O_2 < 0.1 ppm).

2 – Electrochemical cell preparation

$LiClO_4$, THF and ethanol were used to make electrolytes of 0.6 M $LiClO_4$ with varying ethanol content. The THF and ethanol were used as purchased. The $LiClO_4$ was dried under vacuum at 100°C for at least 12 hours.

For investigation of the Hydrogen Evolution Reaction (HER)/Hydrogen Oxidation Reaction (HOR) redox couple, a 1 cm^2 Pt foil attached to a Pt wire was used as the working electrode. A Pt mesh attached to a Pt wire of surface area larger than 1 cm^2 was used as the counter electrode. A Pt wire was used as a pseudo-reference. All Pt electrodes were electropolished in 70% nitric acid at least 1 V vs the counter electrode (a Pt mesh) for 2 minutes. After electropolishing, the electrodes were rinsed in ultra-pure water (>18.2 MΩ, Sartorius) and flame annealed.

For nitrogen reduction experiments, the working electrode was switched to a 1 cm^2 Mo foil attached to a Cu wire current collector. These electrodes were dipped in 4M HCl, rinsed in ethanol, polished using 400, 1500 and 2500 grit silicon carbide paper to a mirror finish and then sonicated in ethanol.

The single compartment glass cell was first assembled with the three platinum electrodes for the HER/HOR redox investigation with the working and counter approximately 1 cm apart. The Pt pseudo-reference was placed in-between the two. The cell and Mo electrode were then taken into the Ar glovebox and filled with 15 ml of electrolyte. An 800 µl sample of blank electrolyte was taken for ammonia quantification. The cell was then connected into a closed gas line capable of switch between Ar, N_2 and H_2 gas feeds. The Ar and N_2 gas streams were passed through separate purifiers for inerts upstream of the experiment. A PTFE coated magnetic stirrer was used to agitate the electrolyte.

After electrochemistry, the cell was disassembled inside the glovebox. The electrolyte was sampled for ammonia quantification. The glass cell, rubber stoppers, magnetic stirrer, Pt wire pseudo-reference and Pt mesh counter electrode were removed from the glovebox and boiled in ultra-pure water (>18.2 MΩ, Sartorius) for one hour. The Mo electrode was cleaned in 4M HCl to remove any plated lithium and SEI species. The glass cell, stoppers and Pt electrodes were stored in a glass drying oven at 70°C. The Mo electrode was stored in air.

3 – Electrochemical testing

All experiments were carried out at ambient temperature and pressure. All PEIS spectra were collected between 200kHz and 200mHz about OCV at an amplitude of 10 mV.

3a – First HER/HOR redox (before nitrogen reduction)

The cell was first purged with H_2 gas at 10 ml/min for 10 minutes and the Open Circuit Voltage (recorded). The OCV was observed to settle close to 0 V vs Pt for both electrodes after approximately 5 minutes. If the OCV was too far away from 0 V (± 60 mV), the cell was dissembled and the platinum electrodes cleaned again. The gas flow was then changed to 5 ml/min to avoid too much electrolyte evaporation, and an impedance spectrum taken at OCV to determine the uncompensated resistance. This value was used to correct the potential for the HER/HOR redox. The impedance of the counter electrode is also taken during this measurement. 10 Cyclic Voltammograms (CVs) were then taken at 20 mV s^{-1} from -0.1 V 0.1 V vs OCV. An impedance spectrum was taken again to check for any changes in uncompensated resistance.

3b – Purge and electrode swap

The cell was then purged with Ar gas at 50 ml/min for 10 minutes to remove any hydrogen gas in the cell. The cell was then disassembled and the Pt working electrode replaced with the Mo working electrode. The Pt electrode was stored in a vial in the glovebox. The cell was then re-assembled and purged with N_2 at 10 ml/min for 1 hour to remove any dissolved H_2 in the electrolyte and saturate with N_2. During this time the OCV was recorded to note any changes.

3c – Nitrogen reduction experiments.

Once a stable OCV was observed, an impedance spectrum at OCV was taken to determine the new uncompensated resistance due to any changes in electrode position. This value was used to correct the voltages during the nitrogen reduction regime. Again, the counter electrode impedance is also measured during this technique. A linear sweep voltammogram (LSV) was taken at 20 mV/s until lithium plating was clearly seen. A constant current density of -2 mA cm^{-2} is then applied until -5 C of charge is passed (chronopotentiometry, CP). A second impedance spectrum was is taken at OCV, which was now the lithium plating potential, to determine the SEI impedance.

3d – Purge, sample taking and electrode swap

The cell was then purged with Ar gas at 50 ml/min for 10 minutes to remove any nitrogen gas in the cell. The cell was then disassembled. The volume of the electrolyte was measured via pipetting and 6 x 400 µl samples of electrolyte taken for ammonia quantification. The Mo electrode was replaced with the same Pt electrode as for the initial tests. No further cleaning of the electrode was needed since any dried electrolyte on the Pt electrode immediately redissolved upon re-immersion into the electrolyte. The cell was re-assembled and again purged with H_2 for 10 minutes at 10 ml/min. The OCV was measured and again observed to settle close to 0 V vs Pt for both electrodes after approximately 5 minutes. For the H_2 measurements after nitrogen reduction, the OCV was seen to stabilise within ± 10 mV of 0 V vs Pt.

3e – Second HER/HOR redox

The gas flow was then changed to 5 ml/min to avoid too much electrolyte evaporation, and an impedance spectrum taken at OCV to determine the uncompensated resistance. This value was used to correct the potential for the HER/HOR redox. The impedance of the counter electrode is also taken during this measurement. 10 Cyclic Voltammograms (CVs) were then taken at 20 mV s^{-1} from -0.1 V 0.1 V vs OCV. An impedance spectrum was taken again to check for any changes in uncompensated resistance. The cell was then purged with Ar at 50 ml/min for 10 minutes to remove any H_2 gas in the cell. The cell was disassembled and remaining electrolyte either stored or disposed of.

4 – HER/HOR determination

The HER/HOR redox potential was determined for each IR corrected H_2 CV taken using the below formula

$$U_{HER/HOR} vs\ Pt = \frac{1}{2}\left(\frac{V_-{}^{ox}+V_+{}^{ox}}{2}+\frac{V_-{}^{red}+V_+{}^{red}}{2}\right), \quad (1)$$

where $V_-{}^{ox/red}$ is the potential just below 0 current on the oxidative or reductive sweep and $V_+{}^{ox/red}$ is the potential just above 0 current on the oxidative or reductive sweep. The value for each CV was then averaged across the 10 CVs taken. The standard error in the mean did not exceed 0.9 mV.

5 - Ammonia quantification

The colorimetric indophenol method was used, with a method adapted from that of Lazouski et al.[1], Suryanto et al.[2], and Andersen et al.[3].

Alkaline solution: 800 mg of sodium hydroxide (VWR pellets) was dissolved in 50 ml ultra-pure water to obtain 0.4 M NaOH. The solution was stored at 4°C in the dark, along with the sodium hypochlorite solution. Just before quantification, NaOH was mixed with the stock sodium hypochlorite solution in a 9:1 ratio to obtain approximately 1% sodium hypochlorite.

The phenol nitroprusside catalyst solution was used as purchased.

Sample preparation: Between 5 and 6 samples of 400 µl of the electrolyte were collected depending on electrolyte evaporation and removed from the glovebox along with 800 µl of blank electrolyte (2 x 400 µl) samples. For every 400 µl of sample, 20 µl of 4 M HCl was added. The samples were then evaporated in a water bath between 65 and 70 °C until only a dry residue was obtained (approximately 30 minutes).

Blank samples: The blank samples were each dissolved in 2 ml ultrapure water to obtain 2 x 2 ml samples. These were transferred to cuvettes. One sample was further diluted to 3 ml to act as a baseline.

Used electrolyte samples: The samples were each dissolved in 1.5 ml ultrapure water to obtain 6 x 1.5 ml samples. These were transferred to cuvettes. 20 µl, 15 µl and 10 µl of a solution of known NH_4Cl concentration in ultra-pure water (500 ppm) was added to three of the samples. If a 6th sample was collected, 25 µl of the standard addition solution was added. All samples were then further diluted to 2 ml with ultra-pure water. One sample was further diluted to 3 ml to act as a baseline.

Indophenol method: 500 µl of the alkaline solution was added to all 2 ml samples, followed quickly by 500 µl of the phenol nitroprusside catalyst solution. The cuvettes were agitated slightly to ensure that the solutions were well mixed, and then were covered and left in the dark for 30 minutes.

UV-vis spectroscopy: The absorbance of the samples was measured by UV-vis spectroscopy between 400 nm and 900 nm. The absorbance peak at approximately 650 nm was corrected with the absorbance at the trough (at 900nm) to account for any discolouration of the THF.

Standard addition calculation: The ammonia content in the electrolyte was then evaluated by the standard addition method as described by Suryanto et al.[2]. To account for any (negligible) unavoidable ammonia in the samples, which can come from multiple sources in the lab despite our rigorous cleaning protocol[3], the absorbance of the blank sample was taken away from the absorbance of all the others.

Faradaic efficiency calculation: The Faradaic efficiency was calculated using the below equation,

$$FE_{NH3}\ (\%) = \frac{3\ F\ C_{NH3} V}{Q},$$

where F is the Faraday constant, C_{NH3} is the concentration of ammonia as measured by the salicylate method, V is the volume of electrolyte as measured at the end of the experiment and Q is the charge passed.

Contamination testing: To ensure that the hydrogen measurements before the nitrogen reduction experiment did not introduce contamination, Ar and N_2 blanks were carried out as per the protocol laid out by Andersen et al[3]. Given that the $LiClO_4$ system has already been verified by the same authors, isotopically labelled measurements were not carried out. An Ar blank consists of carrying out the same electrochemistry, but without introducing N_2, to ensure that there are no contaminants in the cell. An N_2 blank consists of carrying out the hydrogen measurements but leaving the cell at OCV for approximately 2 hours under the flow of nitrogen to ensure there are no contaminants in the gas stream. The 1 vol % ethanol condition was tested since both the 0.5 and 1 vol % conditions produced similar amounts of ammonia, whereas the 5 vol % ethanol condition resulted in a much lower Faradaic efficiency. Therefore, if no ammonia is produced in the 1 vol % ethanol condition in blank tests, then the other conditions will also be verified. Since blanks were taken for every experiment as part of the quantification process and all had reproducibly negligible absorbance, we are confident that the

electrolyte itself is not a source of contamination. Both samples yielded negligible ammonia compared to the active condition, as shown in Figure S6.

6 – Energy efficiency calculations

The energy efficiency of the system was calculated using the same method as Fu and Pedersen et al[4]. Here, the energy efficiency is defined as the ratio of useful energy out to energy in. The useful energy out is taken to be the free energy of ammonia oxidation to water ($\Delta G_r{}^0$ = 339 kJ mol^{-1}) multiplied by the number of moles of ammonia produced (n_{NH_3}):

$$Energy\ efficiency = \frac{E_{out}}{E_{in}} = \frac{\Delta G_r{}^0 \cdot n_{NH_3}}{\int V_{cell}(t) \cdot I(t)dt}$$

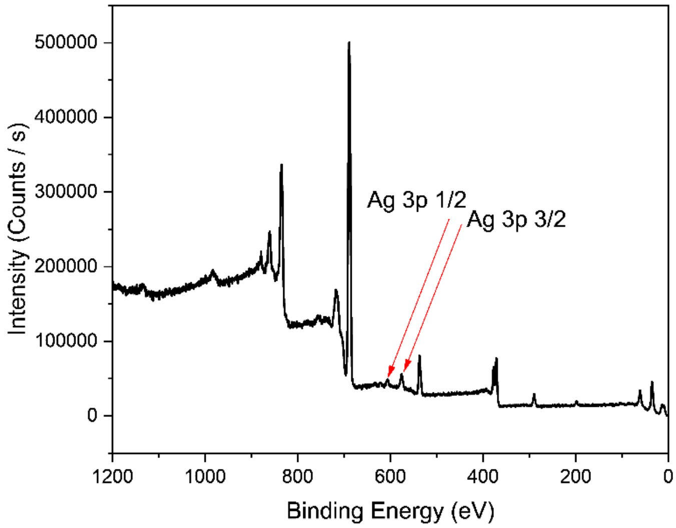

Fig S1: An X-Ray Photoelectron Spectroscopy survey spectrum taken of an electrode after a nitrogen reduction experiment in a 0.2 M $LiBF_4$ electrolyte. Peaks for Ag 3p ½ and 3p 3/2 can be clearly identified, suggesting that the Ag wire pseudo reference was not stable under the nitrogen reduction operating potential.

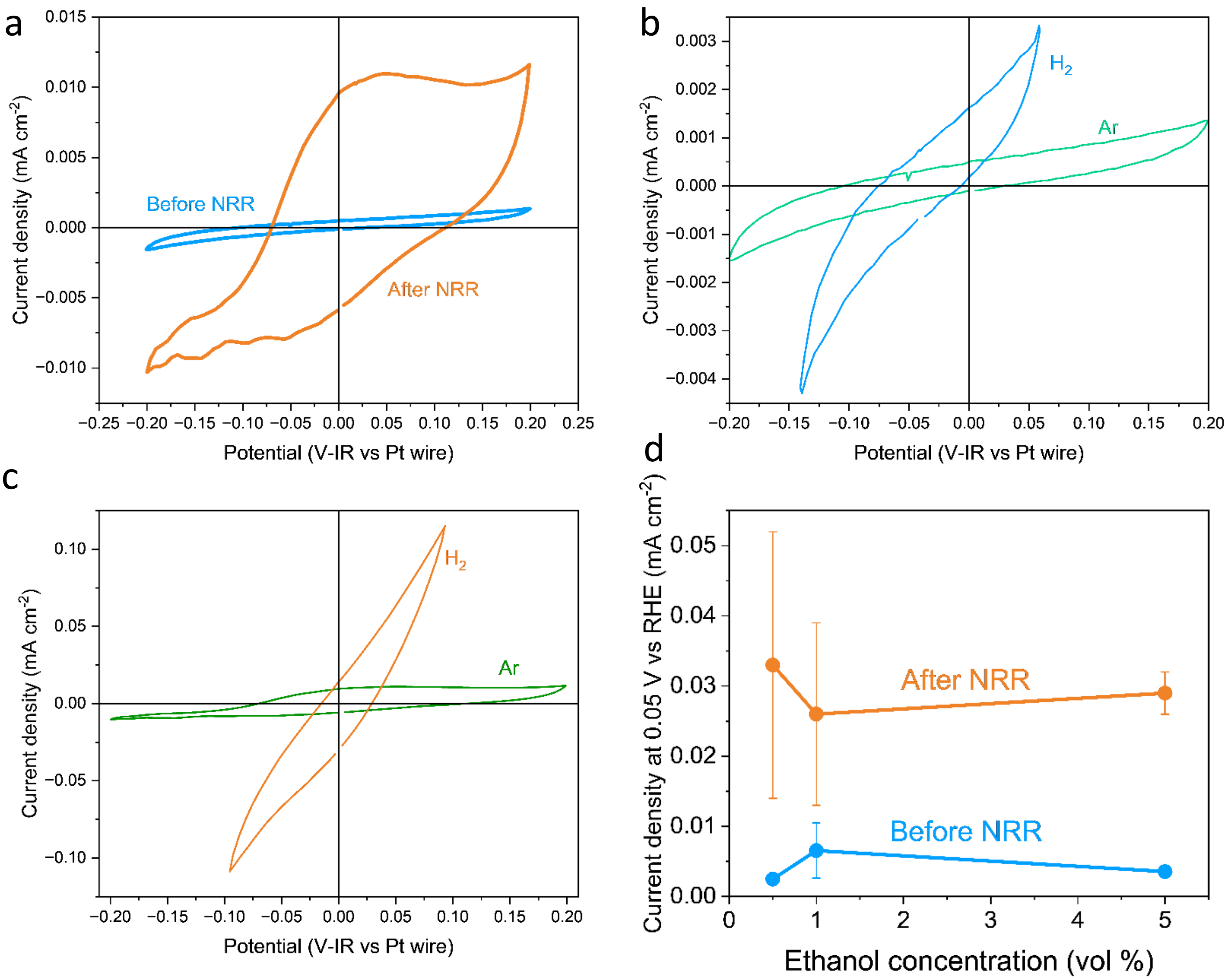

Fig S2: (a-c) All CVs taken at 20 mV/s. 5th cycle shown in 0.6 M LiClO4 99:1 THF:EtOH electrolyte. (a) The change in activity around 0 V vs Pt wire in Ar before and after nitrogen reduction. (b) A comparison between the activity around 0 V vs Pt wire in Ar and in H_2 before nitrogen reduction. (c) A comparison between the activity around 0 V vs Pt wire in Ar and H_2 after nitrogen reduction. (d) A plot to show the change in current density at 0.05 V vs RHE in H_2 taken before and after a nitrogen reduction experiment. There is no significant change in activity with ethanol concentration, but all experiments show and increase in activity before and after nitrogen reduction. (n=3)

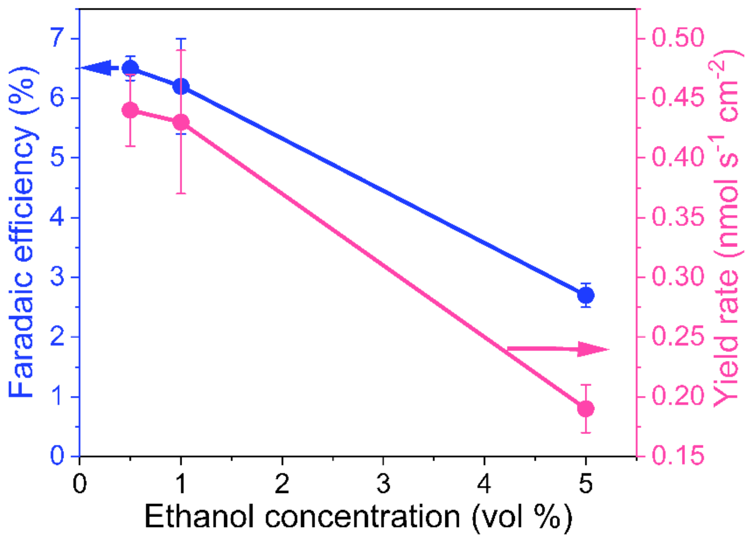

Fig S3: A plot to show the variation in Faradaic efficiency toward nitrogen reduction with ethanol concentration (n=3).

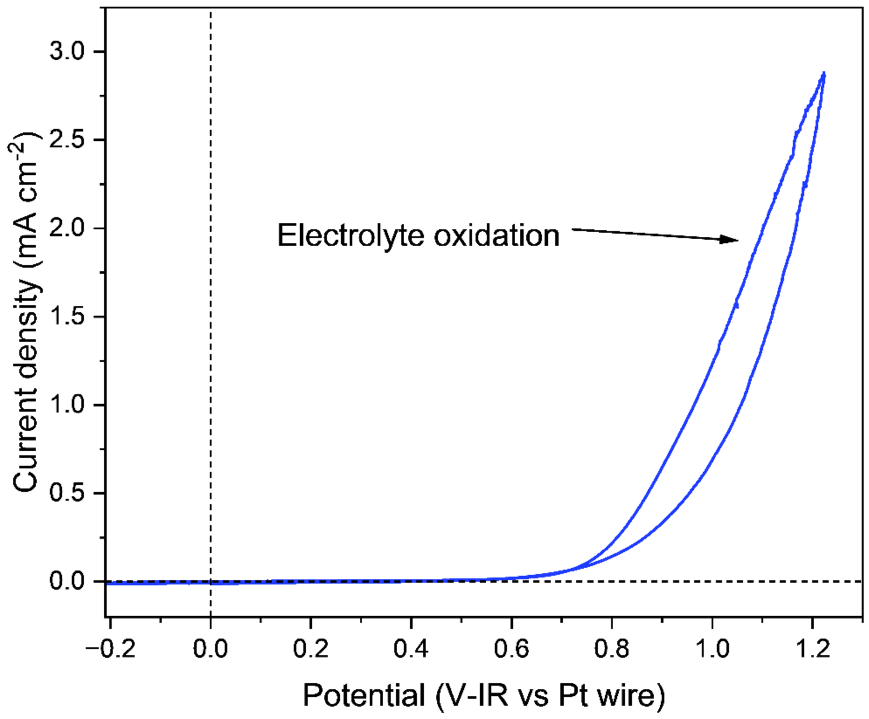

Fig S4: A CV taken in argon showing solvent oxidation occurring at potentials higher than 0.6 V vs the Pt wire. The electrolyte was 0.6 M $LiClO_4$ in 1 vol % ethanol in THF.

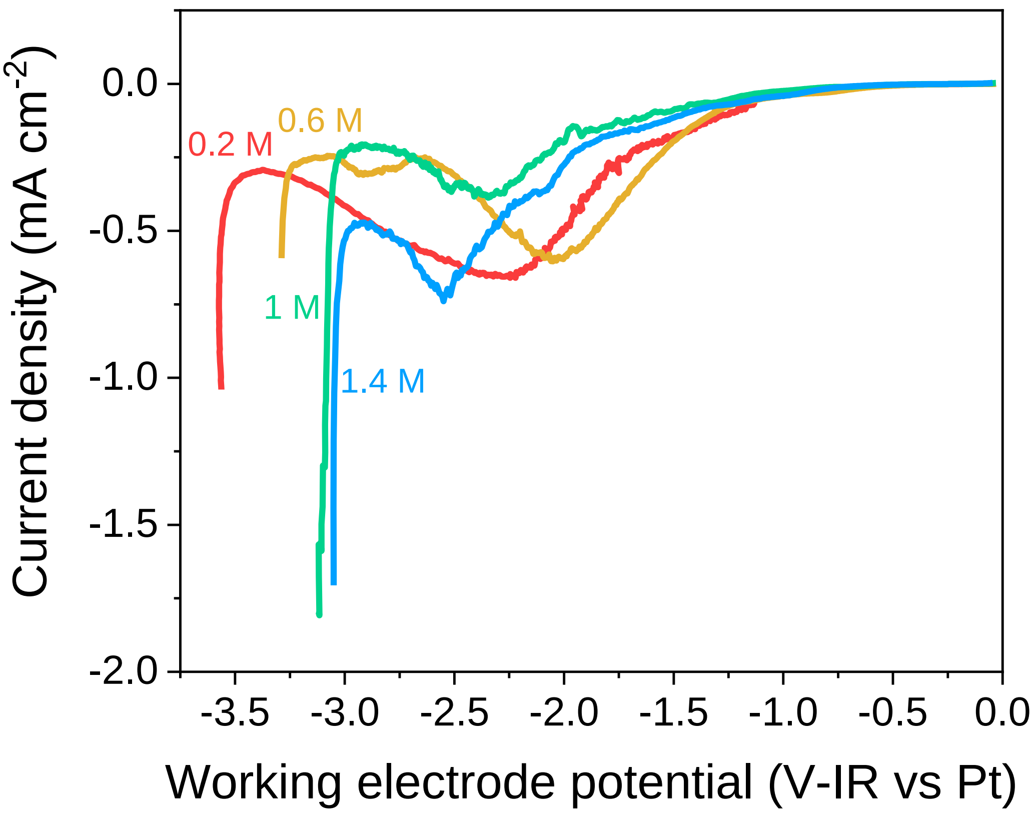

Fig S5: A plot to show the change in lithium plating potential with LiClO4 concentration against the Pt pseudo-reference

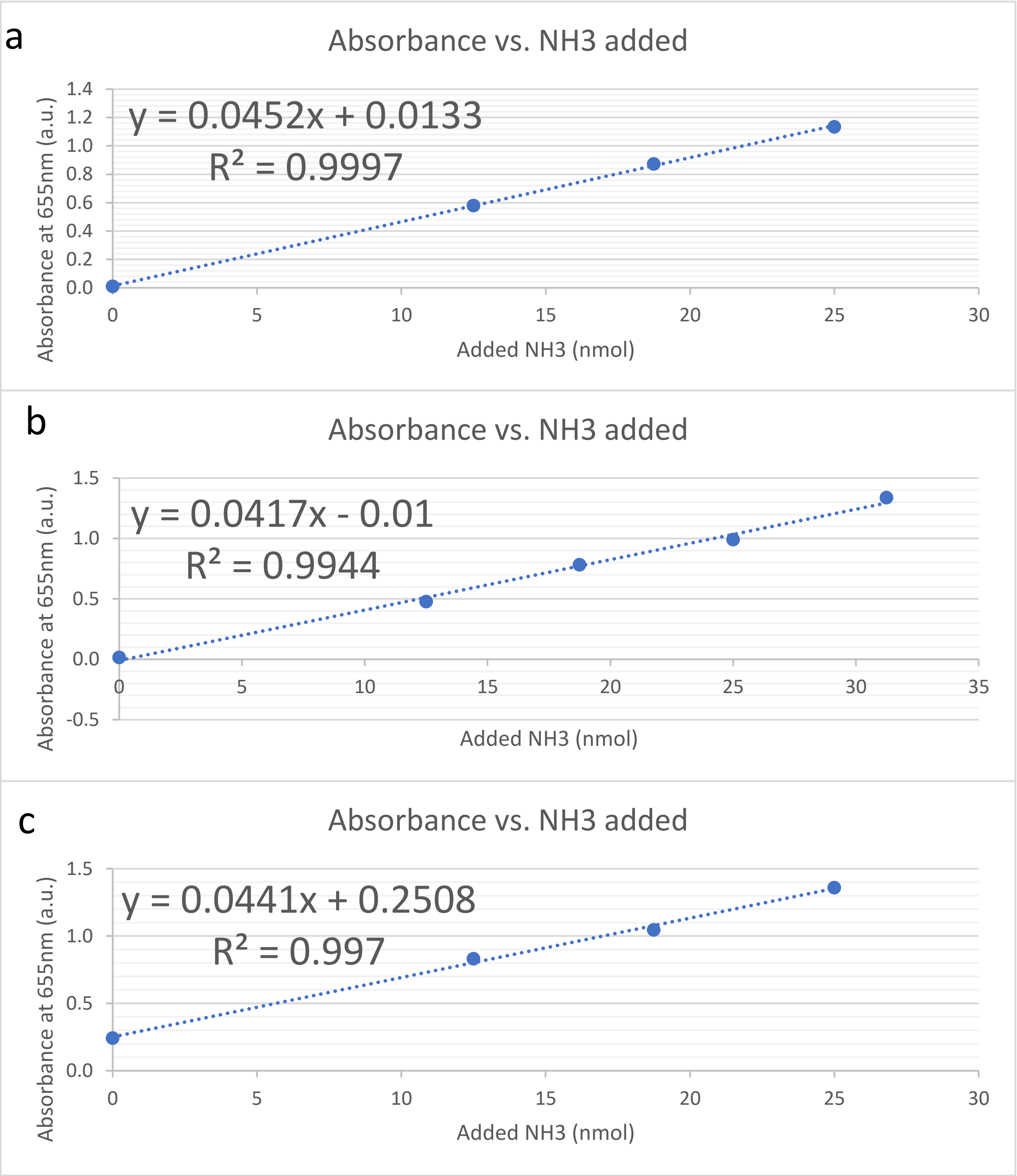

Fig S6: Standard addition results for the quantification of the (a) Ar blank and (b) N_2 blank measurements taken using a 0.6 M $LiClO_4$, 1 vol% ethanol in THF electrolyte. Negligible ammonia was produced in both conditions. (c) shows a representative standard addition result for a 0.6 M $LiClO_4$, 1 vol% ethanol in THF electrolyte experiment where ammonia was made (here, the Faradaic efficiency was 7.7 ± 0.9 % and yield rate was 0.53 ± 0.06 nmol s^{-1} cm^{-2}). The absorbance of the sample measured in (c) is two orders of magnitude larger than in the blank condition.

References

1. Lazouski, N., Schiffer, Z. J., Williams, K. & Manthiram, K. Understanding Continuous Lithium-Mediated Electrochemical Nitrogen Reduction. *Joule* **3**, 1127–1139 (2019).

2. Suryanto, B. H. R. *et al.* Nitrogen reduction to ammonia at high efficiency and rates based on a phosphonium proton shuttle. *Science* **372**, 1187–1191 (2021).

3. Andersen, S. Z. *et al.* A rigorous electrochemical ammonia synthesis protocol with quantitative isotope measurements. *Nature* **570**, 504–508 (2019).

4. Fu, X. *et al.* Continuous-flow electrosynthesis of ammonia by nitrogen reduction and hydrogen oxidation. *Science* **379**, 707–712 (2023).
